# Handheld Microflow Cytometer Based on a Motorized Smart Pipette, a Microfluidic Cell Concentrator, and a Miniaturized Fluorescence Microscope

**DOI:** 10.3390/s19122761

**Published:** 2019-06-19

**Authors:** Byeongyeon Kim, Dayoung Kang, Sungyoung Choi

**Affiliations:** Department of Biomedical Engineering, Kyung Hee University, 1732 Deogyeong-daero, Giheung-gu, Yongin-si, Gyeonggi-do 17104, Korea; kby921022@khu.ac.kr (B.K.); dayoung365@khu.ac.kr (D.K.)

**Keywords:** microflow cytometry, smart pipette, hydrophoresis, miniaturized fluorescence microscope, microfluidics, residual white blood cells

## Abstract

Miniaturizing flow cytometry requires a comprehensive approach to redesigning the conventional fluidic and optical systems to have a small footprint and simple usage and to enable rapid cell analysis. Microfluidic methods have addressed some challenges in limiting the realization of microflow cytometry, but most microfluidics-based flow cytometry techniques still rely on bulky equipment (e.g., high-precision syringe pumps and bench-top microscopes). Here, we describe a comprehensive approach that achieves high-throughput white blood cell (WBC) counting in a portable and handheld manner, thereby allowing the complete miniaturization of flow cytometry. Our approach integrates three major components: a motorized smart pipette for accurate volume metering and controllable liquid pumping, a microfluidic cell concentrator for target cell enrichment, and a miniaturized fluorescence microscope for portable flow cytometric analysis. We first validated the capability of each component by precisely metering various fluid samples and controlling flow rates in a range from 219.5 to 840.5 μL/min, achieving high sample-volume reduction via on-chip WBC enrichment, and successfully counting single WBCs flowing through a region of interrogation. We synergistically combined the three major components to create a handheld, integrated microflow cytometer and operated it with a simple protocol of drawing up a blood sample via pipetting and injecting the sample into the microfluidic concentrator by powering the motorized smart pipette. We then demonstrated the utility of the microflow cytometer as a quality control means for leukoreduced blood products, quantitatively analyzing residual WBCs (rWBCs) in blood samples present at concentrations as low as 0.1 rWBCs/μL. These portable, controllable, high-throughput, and quantitative microflow cytometric technologies provide promising ways of miniaturizing flow cytometry.

## 1. Introduction

Flow cytometry is a high-throughput cell phenotyping technology in which a large population of single cells in suspension can be examined as they pass through a region of interrogation (ROI) [1,2,3]. It has become a fundamental tool in numerous cellular applications such as immune cell phenotyping [4,5], transfection [6,7], apoptosis [8], and cell cycle analysis [9,10]. Miniaturizing flow cytometry has great potential in cell biology and diagnostic applications [11,12], but the difficulty of reducing the system size and complexity while retaining its original functionalities has limited its widespread adoption. The miniaturized and handheld format of flow cytometry can be ideal for routine use and will be advantageous in further expanding its applications. In the case of cell counters based on the Coulter principle, a handheld type similar to a micropipette has been developed and commercialized [13,14]. The utility of the portable cell counting technology has been explored as a point-of-care tool by directly counting infectious agents in blood [15]. However, the analytical capability of the handheld cell counter is limited to measuring cell size, making it difficult to apply this to the various biological and diagnostic applications listed above. 

Considerable effort has been invested in miniaturizing flow cytometry that could be potentially handheld, and these can be categorized into the optical approach, to make the optical system of flow cytometry portable, and the fluidic approach, to simplify the fluidic system of flow cytometry [2,11,16,17,18,19,20,21,22,23,24,25,26,27,28,29,30]. Optical technologies have demonstrated the potential of microflow cytometry for mobile healthcare by incorporating a smartphone into a flow cytometer to use its integrated imaging sensor and its computing and communication capabilities [16], and researchers have utilized simplified, lens-free optical systems based on holographic imaging for flow cytometric analysis [17]. In addition, miniaturized fluorescence microscopes have been employed for microflow cytometric applications by compactly integrating a light source, optical filters, an objective lens, and an imaging sensor [18,19]. Although the optical performance of the microflow cytometers is relatively low compared to conventional flow cytometry, these microflow cytometers have been successfully demonstrated to suit specific applications.

The fluidic technologies for miniaturizing flow cytometry have improved the fluidic system of flow cytometry by replacing its conventional sheath-based fluidic chamber with sheathless microfluidic devices or by employing portable pumping mechanisms. The conventional fluidic structure of flow cytometry has been simplified to be driven only by injecting a sample fluid without sheath fluids and controlling cell positions in suspension based on the microfluidic principles of inertial force-based cell focusing [20,21,22], viscoelasticity-based cell focusing [23,24], hydrophoresis [25,26], dielectrophoresis [27], and acoustophoresis [28]. In addition, non-mechanical pumping methods such as electrokinetic and hydrostatic pressure-based pumping techniques have been applied to flow cytometric applications with the advantages that they can be easily miniaturized and fabricated [29,30,31]. Various research efforts have been made to miniaturize flow cytometry, but few techniques have fully miniaturized flow cytometry by simplifying both fluidic and optical systems. 

**Figure 1:**
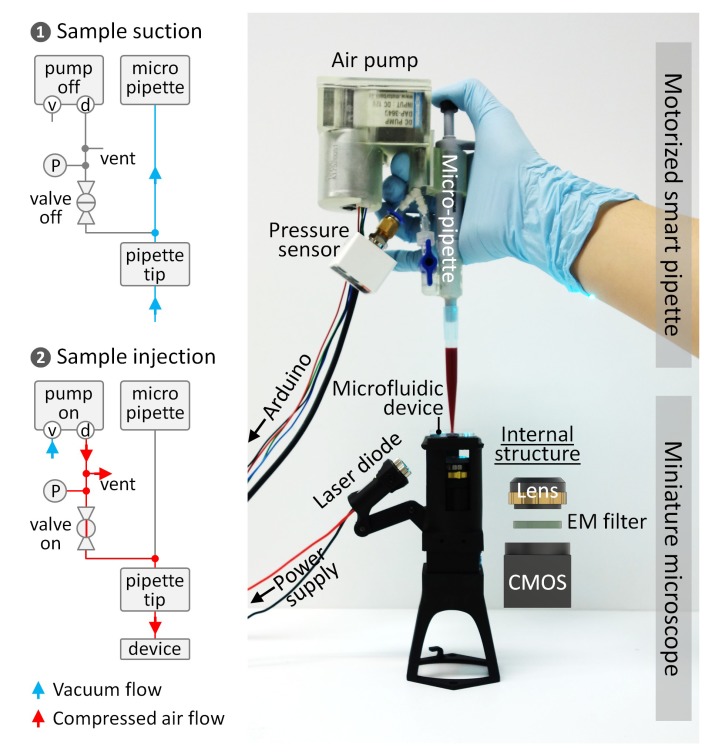
Handheld microflow cytometer composed of a motorized smart pipette for volume metering and pressure control, a microfluidic cell concentrator for target cell enrichment, and a miniaturized fluorescence microscope for imaging flow cytometric analysis. The schematics show the pneumatic control diagrams for smart pipetting. The symbols v, d, and P denote the vacuum and discharging ports of the air pump and a pressure sensor, respectively.

Here, we present comprehensive integration of a motorized smart pipette (i.e., a micropipette-type device for handheld operation of microfluidic devices), a miniaturized fluorescence microscope for portable analysis of fluorescently-labelled cells, and a microfluidic cell concentrator for rare cell analysis, thus achieving the complete miniaturization of flow cytometry and portable detection of residual white blood cells (rWBCs) in blood products (Figure 1). The brand-new smart pipette combines a miniature air pump and a fixed-volume micropipette, thereby allowing simple flow-rate control using a feedback control method and accurate volume metering for the detection of low concentrations of rWBCs, respectively. The miniaturized microscope compactly integrates all optical components for the portable detection of fluorescent objects. The microfluidic concentrator enriches rWBCs at low concentrations to a measurable concentration, thus enabling sensitive cell counting at a high-throughput scale. We demonstrated the capability of the integrated approach for a microflow cytometric application, rWBC counting, by operating the microfluidic concentrator using the smart pipette and simultaneously counting rWBCs using the miniaturized microscope. Using the integrated microflow cytometer, we successfully measured low concentrations of rWBCs as low as 0.1 rWBCs/μL at a high throughput of about 370.4 μL/min, successfully demonstrating its potential for handheld flow cytometric applications.

## 2. Materials and Methods 

### 2.1. Device Design and Fabrication

The motorized smart pipette was fabricated by compactly assembling a miniature air pump (Motorbank Corp., Gimpo-si, Korea), a fixed-volume pipette (Microlit Corp., Paikaramau, India), a pressure sensor (Autonics Corp., Busan, Korea), a digital I/O board (Arduino Uno Rev3), a three-way connector (isupply Corp., Seongnam-si, Korea), and a two-way valve (isupply) in a 3D-printed housing (Figure 1). The discharging port of the air pump was connected directly to the perforated hole on the side of the fixed-volume pipette through a three-way connector and a two-way valve. They were all connected using tubing pieces with an inner diameter of 4 mm. The remaining port of the three-way connector was connected to the pressure sensor. One of the tubing pieces was punched using a biopsy punch with a diameter of 1 mm to generate a vent hole. The two-way valve could be set to block the connection of the discharging port to the fixed-volume pipette to draw up a liquid sample, or to permit the connection to operate a microfluidic device. 

The microfluidic concentrator was fabricated by two-step photolithography and polydimethylsiloxane (PDMS) replica molding as described previously [18]. The photoresist (PR) mold for the concentrator was fabricated on a silicon wafer by repeating photolithography processes including PR spin-coating, soft baking to evaporate solvent, ultraviolet exposure through a mask (Microtech Corp., Ansan-si, Korea), hard baking to solidify the resulting PR structures, and development to remove the unexposed PR areas. The first PR layer, which was 23 µm high, was defined as a microfluidic channel network to connect separation channels in series and in parallel. The second PR layer, which was 28 µm high, was patterned to stack slanted ridges on the channel network. The PR patterns were then transferred to PDMS by the thermal curing of a mixture of PDMS and a curing agent (Dow Corning, Inc., Midland, MI, USA) at a 10:1 mass ratio on the PR mold. The PDMS replicas were then punched for inlet and outlet holes, cut into individual devices, and irreversibly sealed with a glass slide after air plasma treatment. 

The miniaturized fluorescence microscope was fabricated by compactly integrating a laser diode (488 nm; DTR Laser, St. Louis, MO, USA), a longpass filter with a cut-on wavelength of 515 nm (Omega Optical, Inc., Brattleboro, VT, USA), a 2× objective lens (Edmund Optics, Inc., Barrington, NJ, USA), and a CMOS sensor (FLIR Systems, Inc., Wilsonville, OR, USA) in a 3D-printed housing. The 3D-printed housing contained a chip mount onto which the microfluidic concentrator could be firmly fixed via bolt tightening, thus preventing cell images from being out of focus due to pumping vibrations. 

### 2.2. Sample Preparation

Glycerol was purchased from Sigma-Aldrich, Inc. (St. Louis, MO, USA) and diluted in deionized water (Welgene Corp., Gyeongsan-si, Korea) at a concentration range of 45.5 to 68.5% (wt/wt) to characterize the volume-metering performance of the smart pipette. The volume of a liquid drawn up into a 1-mL pipette tip was determined by measuring the liquid weight and multiplying it by the liquid density. Canine blood samples were obtained from the Korea Animal Blood Bank (Sokcho-si, Korea) and centrifuged to generate WBC-depleted blood samples by removing a buffy coat layer. The blood samples were then filtered with a WBC syringe filter (Pall Corp., Post washington, NY, USA) to completely remove the remaining WBCs. Then, the sorted WBCs were stained with a cell-permeant SYTO13 nucleic acid stain (Thermo Fisher Scientific, Inc., Waltham, MA, USA), counted using a hemocytometer, and spiked into the WBC-depleted blood samples at the desired concentrations to generate model rWBC samples. The final hematocrit of the blood samples was about 22%. A bench-top fluorescence microscope (Nikon Corp., Minato, Japan) was used only for taking device pictures, and a commercial syringe pump (KD Scientific Inc., Holliston, MA, USA) was used only for the characterization of the microfluidic concentrator. The miniaturized microscope and the smart pipette developed here were used for all other experiments.

### 2.3. Experimental Setup

A custom Arduino-based feedback controller was developed using a feedback loop in which the pulse-width modulation (PWM) duty cycle for driving the air pump was modulated depending on the pressure value measured using the pressure sensor by increasing the duty cycle when the measured pressure value was below a set pressure and vice versa. The reference duty cycles for the feedback control were 37, 43, 49, and 55% at 20 kHz for the set pressures of 80, 105, 135, and 150 kPa, respectively. Automated cell counting software was developed using a library of programming functions for open-source computer vision (OpenCV), as described previously [18]. Cell images extracted from a fluorescence video taken using the miniaturized microscope were processed for noise reduction by sequentially applying multiple functions and algorithms such as a Gaussian blur, a background subtraction, and the morphologyEx function. In the processed images, single cells were identified using a blob detecting algorithm, the SimpleBlobDetector function with set threshold values for blob size, circularity, and pixel intensity. The cells were then enumerated when they crossed an interrogation area in the vertical direction of the images. 

## 3. Results and Discussion

### 3.1. Volume Metering and Flow Rate Control Using the Motorized Smart Pipette

**Figure 2:**
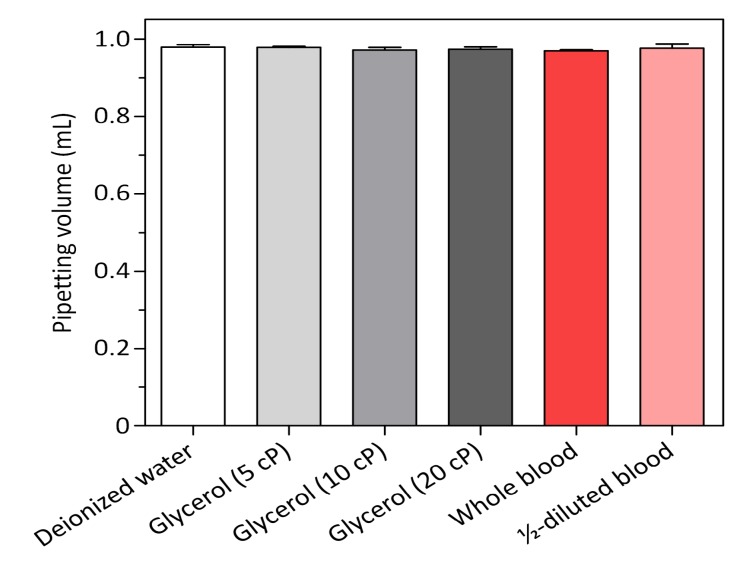
Volume-metering performance of the smart pipette. The embedded fixed-volume micropipette allows the delivery of a precise volume of various liquid samples into a microfluidic device (*n* = 3).

Measuring a sample volume during flow cytometric analysis can allow one to obtain a precise cell count, but typically requires bulky, high-precision pumping and flow-sensing equipment, which make it difficult to miniaturize flow cytometry. To address this challenge, we developed the motorized smart pipette capable of accurate volume metering and simple pressure control, thereby replacing the bulky fluidic equipment necessary for conventional flow cytometry with a handheld device. The smart pipette was fabricated by introducing a fixed-volume micropipette into our previous smart-pipette design for the portable operation of a microfluidic blood plasma separator [32]. Thus, an accurate volume (≈0.97 mL) of a sample fluid can be drawn up into a 1-mL pipette tip via simple pipetting, and then the sample can be injected into a microfluidic device by properly setting the two-way valve and powering the air pump of the smart pipette (Figure 1). The valve controls the direction of air flow, either from the atmosphere to the micropipette or from the air pump to the pipette tip, which prevents pressure leakage during sample drawing and ensures accurate volume metering. When tested with various types of solutions (i.e., deionized water, blood, and viscous liquid), the smart pipette allowed accurate volume metering with a coefficient of variance (CV) of less than 1.2% (Figure 2), demonstrating the capability of the smart pipette to deliver an accurate volume of a liquid sample to a region of interrogation. The sampling volume was less than the nominal micropipette volume of 1 mL, which was likely due to the error of the micropipette itself. 

**Figure 3:**
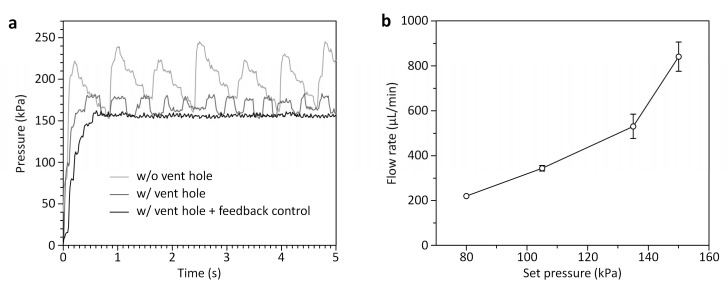
Pressure and flow-rate control using the smart pipette. (**a**) Pressure control responses to a step command for a set pressure of 150 kPa with a duty cycle of 55%. The combination of the vent hole and the feedback control allows stable pressure control with a CV of 1.2%. (**b**) Flow-rate control with set pressures. The flow rate presented here was calculated by dividing the blood volume injected during smart pipetting through the microfluidic cell concentrator by time. Error bars represent standard deviations (*n* = 3).

In addition to the function of liquid metering, the motorized smart pipette provides the capability of controlling air pressure using PWM that had not been achieved with our previous smart pipette due to the instability of compressed air pressure by the blockage of the discharging port of the air pump [32]. If the discharging port is clogged, the air pump repeats the process of stopping when it reaches a pressure limit and re-starting when the pressure becomes lower, thus resulting in a large variance in generated pressure (Figure 3a). Since the changes in pressure and flow rate can affect cell enrichment and cell counting performance, stable pressure generation is important for reliable cell counting. Interestingly, we found that the pumping fluctuation could be reduced by punching a vent hole with a diameter of 1 mm in the smart pipette (Figure 3a). This is likely attributed that the vent hole can work as a pressure buffer to discharge air at a varying rate depending on the generated pressure, thus preventing sudden, irregular increases in pressure due to the port blockage. In addition to the vent hole, the feedback control of the air pump enabled more stable pressure generation with a CV of 1.2%. Using the vented smart pipette and simple feedback control, we demonstrated the capability of the smart pipette to generate a range of pressures from 76.7 to 150.9 kPa, and to operate the microfluidic concentrator in a range of flow rates from 219.5 to 840.5 μL/min (Figure 3b). We noted that there was a non-linear correlation between the set pressures and the generated flow rates. This is likely because blood is a non-Newtonian fluid that can change its viscosity under shear stress. Since blood exhibits a shear-thinning characteristic of decreasing blood viscosity with shear stress, the generated flow rate could increase sharply when the applied pressure increased. 

### 3.2. Microfluidic Cell Concentrator

**Figure 4:**
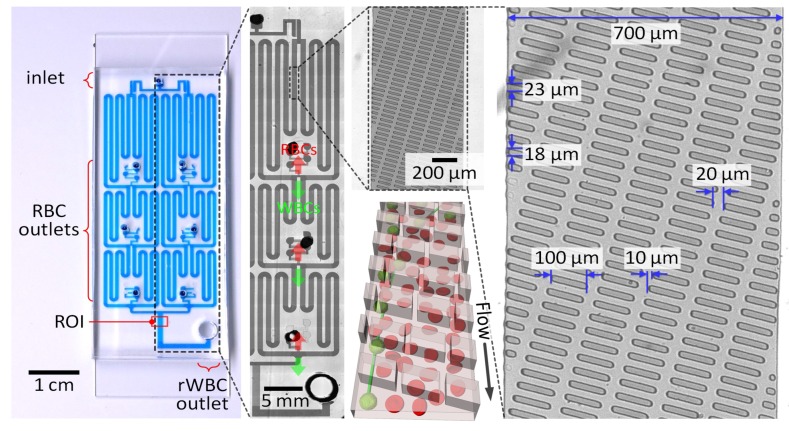
Photographs and schematic (not to scale) showing the microfluidic cell concentrator composed of slanted ridges. Four concentrator units are connected in parallel, and each unit contains three concentration stages in series. The ROI denotes the region of interrogation for rWBC counting.

We used the microfluidic concentrator with a network of slanted ridge-patterned separation channels as a flow chamber for microflow cytometry to passively concentrate and accurately measure low concentrations of rWBCs, as described in our previous work [18]. Briefly, the network consists of four parallelized cell separation units, each of which has three separation channels connected in series (Figure 4). The slant ridges were patterned at a height of 28 μm on the linear channel with a width of 700 μm and a height of 23 μm. The slanted ridges patterned on the separation channels generate rotational streams, and hydrodynamic interactions induced by the rheological properties of red blood cells (RBCs) force WBCs to remain out of the ridges [33,34]. As a result, target WBCs can laterally migrate and be focused at an angle opposite to the inclination angle of the ridges (Figure 4), and they can then be enriched sequentially by discharging a WBC-free RBC stream at the branch of each separation channel. At an optimal flow rate of 400 µL/min, as characterized in our previous work [18], the microfluidic concentrator could ensure effective WBC enrichment by lowering the flow rate through the WBC outlet to ≈4.4 µL/min and thus highly reducing an effective sample volume to be examined 90 times. The microfluidic concentrator can concentrate blood samples containing WBCs instead of completely separating WBCs and RBCs, so that the blood stream to the rWBC outlet still contains RBCs. However, the presence of RBCs did not affect the detection of rWBCs using the microflow cytometer, since RBCs do not have a nucleus and are not stained with the nuclear staining dye. We did not observe WBCs at the RBC outlets, recovering all injected WBCs at the WBC outlet and demonstrating that the microfluidic concentrator can be used for rapid target cell enrichment and reliable cell counting. This enrichment process can result in high-throughput cell counting and cost-effective cell imaging without high-speed and sensitive imaging sensors.

### 3.3. Microscopic WBC Imaging Using the Miniaturized Fluorescence Microscope

**Figure 5:**
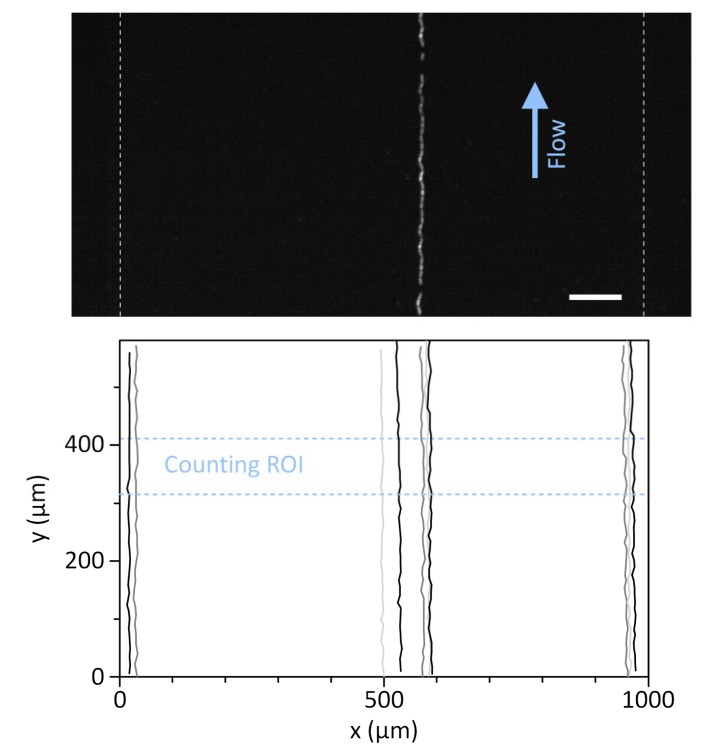
Effect of pumping vibration on cell imaging using the miniature fluorescence microscope. The top and bottom panels show the overlaid fluorescence trajectory of a single WBC and the trajectory plot of 10 independent WBCs, respectively. Single cells were traced through the imaging area by measuring and overlapping their positions in the x-y plane over time. The vibration was generated mainly in the *x*-axis direction, so that the imaged cells appeared to be slightly shaken. However, there was no significant change in the *z*-axis focus of the imaged cells. Scale bar, 100 µm.

**Figure 6:**
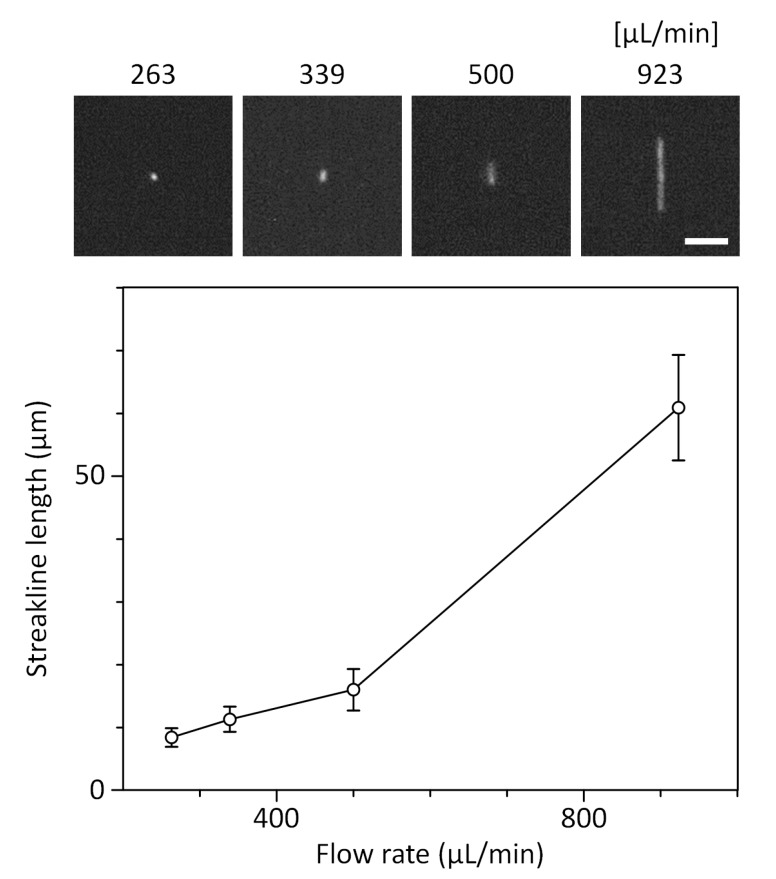
Effect of flow rates on the streakline length of WBC images taken using the miniature fluorescence microscope (*n* = 20 cells for each data point). The flow rates were generated in the microfluidic cell concentrator using the smart pipette with set pressures of 80, 105, 135, and 150 kPa, respectively, in ascending order. Scale bar, 40 µm.

The vibration of the air pump can cause difficulty in microscopic imaging when combined with the miniaturized fluorescence microscope. We thus tested the compatibility of the smart pipette with the miniaturized microscope. The field-of-view of the miniaturized microscope was set to 1.446 mm × 0.579 mm so that the region of interrogation, a microchannel 1 mm in width, could be taken with sufficient margin. We evaluated the degree of vibration as the lateral movement of WBCs flowed along the mainstream flow direction, and as a result, the cells could be shifted within 1.9 ± 1.4 μm from the initial position at the set pressure of 105 kPa and the corresponding flow rate of ≈370.4 μL/min (Figure 5). The effect of the vibration on cell imaging was negligible in comparison to the cell size, so that the vibration did not affect the imaging analysis of rWBCs. Even so, further introduction of vibration dampers will be beneficial for minimizing the effect of vibration. The CMOS sensor of the microscope has a maximum frame rate of up to 150 frames per second for an imaging size of 1280 × 512 pixels. A blood sample can be injected at a high flow rate of 370 μL/min, but the rWBC enrichment process in the microfluidic cell concentrator can reduce the flow rate by more than 90 fold in the ROI by draining unfocused RBC streams (Figure 4). Thus, the resulting velocity of rWBCs flowing through the ROI was 1.39 ± 0.13 mm/sec or 13.9 ± 1.3 μm/frame at the injection flow rate of 370 μL/min (*n* = 10), which was low enough to be measured with the CMOS sensor with a relatively low image acquisition rate of 150 fps. If a cell moves faster than the frame rate, the cell image can exhibit motion blur streaks, which can cause cell analysis to be less accurate. To evaluate the motion blur effect and determine an optimal flow rate condition, we operated the microfluidic concentrator at different flow rate conditions of ≈263.2, 339.0, 500.0, and 923.1 μL/min that were generated using the smart pipette with set pressures of 80, 105, 135, and 150 kPa, respectively. As observed with the miniaturized microscope, pressure conditions below 105 kPa could ensure cell imaging without significant motion blur (Figure 6). The diameter of the fluorescently-stained WBCs was 9.8 ± 1.3 µm (*n* = 40), which was similar to the streakline length of rWBCs flowing in the ROI under pressure conditions below 105 kPa. Thus, we performed the following experiments at an optimal set pressure condition of 105 kPa.

### 3.4. Handheld rWBC Counting Using Integrated Microflow Cytometry

**Figure 7:**
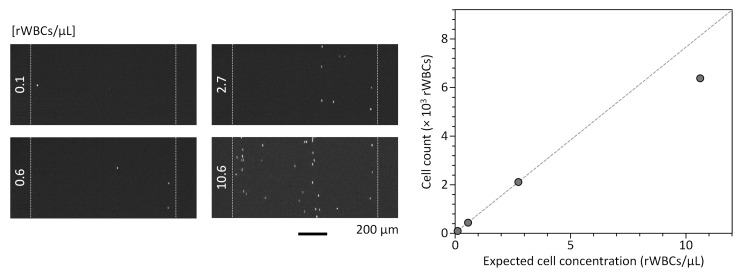
Handheld rWBC counting using the integrated microflow cytometer. Microscopic movies taken using the miniaturized fluorescence microscope were analyzed using the custom cell counting program. Each fluorescence image taken at a specific time point shows the difference in rWBC counts according to the cell concentration. The cell counting results obtained using the microflow cytometer exhibited a linear correlation with the injected rWBC concentrations, which ranged from 0.1 to 2.7 rWBCs/µL. The dotted line represents a linear regression line for the data points. Error bars represent standard deviation (*n* = 3).

We applied the microflow cytometer integrating the motorized smart pipette, microfluidic cell concentrator, and miniaturized fluorescence microscope to rWBC counting. The rWBCs are the remaining WBCs in blood products even after leukoreduction and should be regulated below 1 rWBCs/μL, because they can be a cause of clinical complications such as cytomegalovirus infection, human leukocyte antigen alloimmunization, graft-versus-host disease, and febrile nonhemolytic transfusion reactions [35,36]. However, current methods for rWBC counting rely on bulky, expensive equipment (e.g., flow cytometry and hematology analyzer) or laborious manual counting [37,38]. Microfluidic cell counting methods, which are potentially applicable to rWBC counting, often lack the capacity for high-throughput cell counting [39,40]. We also developed an rWBC counter based on the microfluidic cell concentrator, but this also requires bulky, expensive pumping equipment [18]. With the smart pipette developed here, we could simply draw up a blood sample and operate the microfluidic concentrator by turning on the air pump to achieve handheld and high-throughput operation of the integrated microflow cytometer (Video S1). For rWBC counting, 0.97 mL of rWBC sample was injected into the microfluidic concentrator at a volumetric throughput of ≈370.4 µL/min. The rWBC counting was performed by taking a movie of the enriched WBC streams passing through the ROI located in the front of the WBC outlet using the miniaturized microscope and analyzing it using the custom cell counting program (Video S2). As shown in Figure 7, rWBC counts exhibited a linear correlation with the injected rWBC concentrations (*C*_in_) that ranged from 0.1 to 2.7 rWBCs/µL (*R*^2^ = 0.9999), demonstrating that the integrated microflow cytometry can enable sensitive rWBC counting. The CVs for the rWBC counts were 6.1, 1.7, 3.2, and 1.8% for *C*_in_ = 0.1, 0.6, 2.7, and 10.6 rWBCs/µL, respectively. We note that the data point at *C*_in_ = 10.6 rWBCs/µL showed a large deviation from the linear regression line. This is attributed to the fact that highly enriched rWBCs can flow very close to each other, and thus multiple cells can be recognized as one object by the automatic cell counting program. Although the rWBC count at the high *C*_in_ was inaccurate, the microflow cytometer is suitable for the rWBC counting application, because it shows a linear correlation near the critical concentration range (1 rWBCs/µL) for the quality control of blood products. The linear counting range can be further extended by diluting rWBC samples or adding a counting algorithm for dividing multiple adjacent cells. We also note that there was a relative error of more than 7.7% even within the linear counting range. That is, the cell counts measured using the microflow cytometer were lower than the estimated cell counts. This relative counting error might be due to the remaining cells in the microfluidic concentrator. After 0.97 mL of blood sample was injected into the concentrator, the air began to flow into the concentrator and the volumetric rate of the blood flow increased sharply, resulting in significant motion blur. Although the remaining cells in the concentrator could not be counted under the current operational protocol, there was a strong linear correlation between the measured cell counts and the estimated rWBC concentrations, allowing sensitive detection of low concentrations of rWBCs. The counting error can be corrected by developing an operational protocol to discharge the remaining cells in the cell concentrator without pressure changes and to count all injected cells. 

Although we showed the same application (rWBC counting) using the same microfluidic device used in our previous work [18], the synergetic integration of the major components for microflow cytometry to enable handheld operation is a new approach not previously reported. To achieve this, we developed a new motorized smart pipette capable of volume metering and PWM control, and a miniaturized fluorescence microscope that was lightweight (158 g) and sized for one handed use. The quantitative analysis of nuclear staining for flowing single rWBCs was performed and used for rWBC identification, which meets the functionality of microflow cytometry. However, the current form of the cell concentrator lacks the ability for 3D cell focusing, thus limiting its use to various microflow cytometric applications that require accurate phenotypic analysis. To address this limitation, microfluidic cell focusers can be further added to the ROI of the cell concentrator [20,21,22,23,24,25,26,27,28], thereby precisely aligning cells at the point of interrogation and accurately measuring their phenotypes.

## 4. Conclusions

In summary, we proposed a comprehensive approach that enables the complete miniaturization of the fluidic and optical systems for conventional flow cytometry. The motorized smart pipette embedded with a small, fixed-volume micropipette allows accurate volume metering and flow-rate control, converting conventional bulky fluidic equipment into a portable handheld device. The miniaturized fluorescence microscope compactly assembles all the necessary optical components for imaging flow cytometry, thus enabling handheld microflow cytometric analysis. The microfluidic concentrator as a flow chamber was used to enrich target cells and enable high-throughput cell analysis with relatively low system requirements. By synergistically combining these technologies, we demonstrated that integrated microflow cytometer is capable of counting rWBCs in low concentrations. This work focuses on rWBC counting, but its application can be further extended to various microflow cytometric applications with advantages such as automated and handheld operation, simple and compact setup, and high-throughput cell enrichment.

## Supplementary Materials

The following are available online at https://www.mdpi.com/1424-8220/19/12/2761/s1: Video S1, Handheld operation of the microfluidic concentrator using the motorized smart pipette; Video S2, Automatic rWBC counting using the custom cell counting program.

## References

[1] Steinkamp J.A. (1984). Flow cytometry. Rev. Sci. Instrum..

[2] Stavrakis S., Holzner G., Choo J., deMello A. (2019). High-throughput microfluidic imaging flow cytometry. Curr. Opin. Biotechnol..

[3] Laerum O.D., Farsund T. (1981). Clinical application of flow cytometry: A review. Cytometry.

[4] Craig F.E., Foon K.A. (2008). Flow cytometric immunophenotyping for hematologic neoplasms. Blood.

[5] Finak G., Langweiler M., Jaimes M., Malek M., Taghiyar J., Korin Y., Raddassi K., Devine L., Obermoser G., Pekalski M.L. (2016). Standardizing Flow Cytometry Immunophenotyping Analysis from the Human ImmunoPhenotyping Consortium. Sci. Rep..

[6] Ding X., Stewart M.P., Sharei A., Weaver J.C., Langer R.S., Jensen K.F. (2017). High-throughput Nuclear Delivery and Rapid Expression of DNA via Mechanical and Electrical Cell-Membrane Disruption. Nat. Biomed. Eng..

[7] Sharei A., Zoldan J., Adamo A., Sim W.Y., Cho N., Jackson E., Mao S., Schneider S., Han M.-J., Lytton-Jean A. (2013). A vector-free microfluidic platform for intracellular delivery. Proc. Natl. Acad. Sci. USA.

[8] Wlodkowic D., Skommer J., Darzynkiewicz Z. (2009). Flow cytometry-based apoptosis detection. Methods Mol. Biol..

[9] Song S., Kim M.S., Lee J., Choi S. (2015). A continuous-flow microfluidic syringe filter for size-based cell sorting. Lab Chip.

[10] Choi S., Song S., Choi C., Park J.-K. (2009). Microfluidic self-sorting of mammalian cells to achieve cell cycle synchrony by hydrophoresis. Anal. Chem..

[11] Ateya D.A., Erickson J.S., Howell P.B., Hilliard L.R., Golden J.P., Ligler F.S. (2008). The good, the bad, and the tiny: A review of microflow cytometry. Anal. Bioanal. Chem..

[12] Piyasena M.E., Graves S.W. (2014). The intersection of flow cytometry with microfluidics and microfabrication. Lab Chip.

[13] Tahara M., Inoue T., Miyakura Y., Horie H., Yasuda Y., Fujii H., Kotake K., Sugano K. (2013). Cell diameter measurements obtained with a handheld cell counter could be used as a surrogate marker of G2/M arrest and apoptosis in colon cancer cell lines exposed to SN-38. Biochem. Biophys. Res. Commun..

[14] Johnston G. (2010). Automated handheld instrument improves counting precision across multiple cell lines. BioTechniques.

[15] Bennuru S., Pion S.D., Kamgno J., Wanji S., Nutman T.B. (2014). Repurposed automated handheld counter as a point-of-care tool to identify individuals ‘at risk’ of serious post-ivermectin encephalopathy. PLoS Negl. Trop. Dis..

[16] Zhu H., Mavandadi S., Coskun A.F., Yaglidere O., Ozcan A. (2011). Optofluidic fluorescent imaging cytometry on a cell phone. Anal. Chem..

[17] Moon S., Keles H.O., Ozcan A., Khademhosseini A., Hæggström E., Kuritzkes D., Demirci U. (2009). Integrating microfluidics and lensless imaging for point-of-care testing. Biosens. Bioelectron..

[18] Kim B., Shin S., Lee Y., Um C., You D., Yun H., Choi S. (2019). High-throughput residual white blood cell counter enabled by microfluidic cell enrichment and reagent-containing patch integration. Sens. Actuators B.

[19] Balsam J., Bruck H.A., Rasooly A. (2014). Webcam-based flow cytometer using wide-field imaging for low cell number detection at high throughput. Analyst.

[20] Wang X., Zandi M., Ho C.-C., Kaval N., Papautsky I. (2015). Single stream inertial focusing in a straight microchannel. Lab Chip.

[21] Chung A.J., Gossett D.R., Di Carlo D. (2013). Three dimensional, sheathless, and high-throughput microparticle inertial focusing through geometry-induced secondary flows. Small.

[22] Zhao Q., Zhang J., Yan S., Yuan D., Du H., Alici G., Li W. (2017). High-throughput sheathless and three-dimensional microparticle focusing using a microchannel with arc-shaped groove arrays. Sci. Rep..

[23] Asghari M., Serhatlioglu M., Ortaç B., Solmaz M.E., Elbuken C. (2017). Sheathless Microflow Cytometry Using Viscoelastic Fluids. Sci. Rep..

[24] D’Avino G., Romeo G., Villone M.M., Greco F., Netti P.A., Maffettone P.L. (2012). Single line particle focusing induced by viscoelasticity of the suspending liquid: Theory, experiments and simulations to design a micropipe flow-focuser. Lab Chip.

[25] Choi S., Park J.-K. (2008). Sheathless hydrophoretic particle focusing in a microchannel with exponentially increasing obstacle arrays. Anal. Chem..

[26] Song S., Choi S. (2013). Field-free, sheathless cell focusing in exponentially expanding hydrophoretic channels for microflow cytometry. Cytom. Part A.

[27] Kung Y.-C., Huang K.-W., Chong W., Chiou P.-Y. (2016). Tunnel Dielectrophoresis for Tunable, Single-Stream Cell Focusing in Physiological Buffers in High-Speed Microfluidic Flows. Small.

[28] Kalb D.M., Fencl F.A., Woods T.A., Swanson A., Maestas G.C., Juárez J.J., Edwards B.S., Shreve A.P., Graves S.W. (2017). Line-Focused Optical Excitation of Parallel Acoustic Focused Sample Streams for High Volumetric and Analytical Rate Flow Cytometry. Anal. Chem..

[29] Xuan X., Li D. (2005). Focused electrophoretic motion and selected electrokinetic dispensing of particles and cells in cross-microchannels. Electrophoresis.

[30] Fu A.Y., Spence C., Scherer A., Arnold F.H., Quake S.R. (1999). A microfabricated fluorescence-activated cell sorter. Nat. Biotechnol..

[31] Simonnet C., Groisman A. (2006). High-throughput and high-resolution flow cytometry in molded microfluidic devices. Anal. Chem..

[32] Kim B., You D., Kim Y.-J., Oh I., Choi S. (2018). Motorized smart pipette for handheld operation of a microfluidic blood plasma separator. Sens. Actuators B.

[33] Kim B., Choi Y.J., Seo H., Shin E.-C., Choi S. (2016). Deterministic Migration-Based Separation of White Blood Cells. Small.

[34] You D., Oh S., Kim B., Hahn Y.K., Choi S. (2017). Rapid preparation and single-cell analysis of concentrated blood smears using a high-throughput blood cell separator and a microfabricated grid film. J. Chromatogr. A.

[35] Frey B.M., Furrer-Burger M. (2003). Enumeration of residual cells in leucodepleted blood products: Techniques and pitfalls. Universal Leucodepletion: The European Experience.

[36] Cervia J.S., Wenz B., Ortolano G.A. (2007). Leukocyte reduction’s role in the attenuation of infection risks among transfusion recipients. Clin. Infect. Dis..

[37] Szuflad P., Dzik W.H. (1997). A general method for concentrating blood samples in preparation for counting very low numbers of white cells. Transfusion.

[38] Palmer D.S., Birch P., O’Toole J., Henderson D., Scalia V. (2008). Flow cytometric determination of residual white blood cell levels in preserved samples from leukoreduced blood products. Transfusion.

[39] Lee Y., Kim B., Choi S. (2018). On-Chip Cell Staining and Counting Platform for the Rapid Detection of Blood Cells in Cerebrospinal Fluid. Sensors.

[40] Kim B., Lee Y.J., Park J.G., Yoo D., Hahn Y.K., Choi S. (2017). A portable somatic cell counter based on a multi-functional counting chamber and a miniaturized fluorescence microscope. Talanta.

